# A Comprehensive Review on Sensor-Based Electronic Nose for Food Quality and Safety

**DOI:** 10.3390/s25144437

**Published:** 2025-07-16

**Authors:** Teodora Sanislav, George D. Mois, Sherali Zeadally, Silviu Folea, Tudor C. Radoni, Ebtesam A. Al-Suhaimi

**Affiliations:** 1 Automation Department, Technical University of Cluj-Napoca, 400114 Cluj-Napoca, Romania; george.mois@aut.utcluj.ro (G.D.M.); silviu.folea@aut.utcluj.ro (S.F.); radoni.ca.tudor@student.utcluj.ro (T.C.R.); 2 College of Communication and Information, University of Kentucky, Lexington, KY 40506-0224, USA; szeadally@uky.edu; 3 Vice Presidency for Scientific Research and Innovation, Imam Abdulrahman bin Faisal University, Dammam 31441, Saudi Arabia; ealsuhaimi@iau.edu.sa

**Keywords:** artificial olfaction, electronic nose, food quality, food safety, pattern recognition

## Abstract

Food quality and safety are essential for ensuring public health, preventing foodborne illness, reducing food waste, maintaining consumer confidence, and supporting regulatory compliance and international trade. This has led to the emergence of many research works that focus on automating and streamlining the assessment of food quality. Electronic noses have become of paramount importance in this context. We analyze the current state of research in the development of electronic noses for food quality and safety. We examined research papers published in three different scientific databases in the last decade, leading to a comprehensive review of the field. Our review found that most of the efforts use portable, low-cost electronic noses, coupled with pattern recognition algorithms, for evaluating the quality levels in certain well-defined food classes, reaching accuracies exceeding 90% in most cases. Despite these encouraging results, key challenges remain, particularly in diversifying the sensor response across complex substances, improving odor differentiation, compensating for sensor drift, and ensuring real-world reliability. These limitations indicate that a complete device mimicking the flexibility and selectivity of the human olfactory system is not yet available. To address these gaps, our review recommends solutions such as the adoption of adaptive machine learning models to reduce calibration needs and enhance drift resilience and the implementation of standardized protocols for data acquisition and model validation. We introduce benchmark comparisons and a future roadmap for electronic noses that demonstrate their potential to evolve from controlled studies to scalable industrial applications. In doing so, this review aims not only to assess the state of the field but also to support its transition toward more robust, interpretable, and field-ready electronic nose technologies.

## 1. Introduction

The development of the electronic nose started in the 1960s when researchers began exploring artificial olfaction systems capable of mimicking the human sense of smell. In 1964, Wilkens and Hartman introduced the idea of simulating olfactory processes electronically, contributing to the theoretical groundwork for artificial odor recognition systems [1]. In 1965, Buck et al. explored how chemical compounds could be detected through surface effects on metals and semiconductors, laying the principles that underpin modern gas sensor technology [2]. Around the same time, Dravnieks and Trotter developed a polar vapor detector based on contact potential modulation, an early precursor to volatile organic compounds’ (VOCs) sensing mechanisms [3]. In the early 1980s, Persaud and Dodd proposed a system designed to replicate the mechanisms of human smell perception using an array of gas sensors combined with a pattern recognition algorithm [4]. A few years later, Ikegami and Kaneyasu expanded upon this idea by applying an array of semiconductor gas sensors to distinguish the freshness of food products [5]. The concept of an electronic nose (e-nose) as we know it today is considered to be an intelligent system that integrates an array of chemical or gas sensors with signal processing and pattern recognition mechanisms capable of identifying both simple and complex odors [6,7]. Early e-nose models relied on metal oxide semiconductors and conducting polymer sensors, which, despite their limitations, demonstrated the potential for detecting and discriminating complex odor patterns associated with VOCs. Over time, advancements in sensor technology, nanomaterials, and machine learning have significantly improved the performance of e-nose devices, enabling applications in various industries. Today, modern e-noses incorporate biosensors, artificial intelligence, and miniaturized designs, making them more sensitive, selective, and appropriate for real-world applications.

E-noses play an important role in improving quality control, safety, and efficiency in various sectors, making them an indispensable technological advancement. Studies [8,9] emphasize that e-noses are crucial for the following:

- *Ensuring food quality and safety:* E-noses help detect spoilage, contamination, and adulteration in food products, ensuring freshness and quality control.
- *Advancing medical diagnostics:* E-noses are used in disease detection by analyzing breath, sweat, or urine to identify biomarkers associated with conditions like diabetes, cancer, and infections.
- *Enhancing environmental monitoring:* E-noses detect pollutants, hazardous gases, and air quality changes, aiding in environmental protection and public health.
- *Improving industrial process control:* E-noses help monitor manufacturing processes, detect leaks, and ensure consistent product quality in industries such as pharmaceuticals, perfumes, and beverages.
- *Strengthening security and defense:* E-noses are used in explosive and drug detection, helping in law enforcement and military and border security operations.
- *Boosting agriculture and farming:* E-noses assist in monitoring soil conditions, plant health, and pest infestations by detecting VOCs released by plants to improve crop yields and reduce reliance on harmful pesticides.
- *Ensuring workplace safety:* E-noses help prevent occupational hazards by detecting toxic or flammable gases in industrial and laboratory environments.

### 1.1. Research Questions and Contributions

Ensuring food quality and safety is vital for protecting public health, preventing foodborne illness, reducing food waste, maintaining consumer confidence, and supporting regulatory compliance and international trade. In this context, a timely review of e-noses is essential to capture recent innovations, discuss their advantages and limitations, and summarize the key research directions in order to enhance food quality and safety assurance.

#### 1.1.1. Research Questions

The research questions we explore in this survey include the following:

1. Research Question 1 (RQ1): What are the state-of-the-art research results over the last decade in the field of e-nose systems aimed at food quality and safety?
2. Research Question 2 (RQ2): What lessons have been learned from the design and deployment of e-nose systems in laboratory and industrial settings?
3. Research Question 3 (RQ3): What research gaps exist in the application of e-noses for food quality and safety, and what are the future research directions that we must explore to address these gaps?

#### 1.1.2. Research Contributions

We summarize the main research contributions of this work that address the research questions above as follows:

1. We present an in-depth analysis of research results over the past decade in the e-nose field designed for food quality and safety. We concluded the analysis based on a proposed taxonomy, which we developed through a comprehensive examination of peer-reviewed research papers from three scientific databases. We highlight key technological advances, practical implementations, and performance results obtained across various food sectors.
2. We identify critical lessons learned, such as the importance of e-nose components selection (sensors, signal processing unit, data pattern recognition model) according to the type of food and the need to develop suitable data pattern recognition models, as well as new sensors tailored to food quality and safety assessment.
3. We identify current research gaps, such as the lack of real-world validation and limited sensor sensitivity, and we discuss future research opportunities that will improve the reliability, scalability, and industrial applicability of e-nose technologies in food systems.

#### 1.1.3. Organization of This Paper

We organize the rest of the paper as follows: Section 2 introduces the main components of an electronic nose. Section 3 outlines our review methodology, introduces the final dataset of documents selected for analysis, and presents relevant statistical information. Section 4 analyzes the selected research works based on our proposed taxonomy and discusses lessons learned. Section 5 addresses research gaps and recommends future research directions. Finally, in Section 6, we make some concluding remarks.

## 2. E-Nose Components

The definition of an electronic nose presented in [6] highlights the key components of such a system that work together to detect, analyze, and interpret odors: the sensor array, the signal processing unit, and the pattern recognition system.

The *sensor array* is the core component of the e-nose, comprising multiple chemical or gas sensors that respond differently to various VOCs, creating a unique pattern for different odors. To provide a clearer understanding of sensor technologies, we adopted a classification based on the way each type of sensor operates. Common categories include the following:

- *Chemiresistive sensors*, such as metal oxide semiconductor (MOS) sensors [10] and carbon nanotube (CNT) sensors [11], detect gases through changes in electrical resistance upon exposure to VOCs.
- *Conductometric sensors*, including conducting polymer (CP) sensors [12], alter their conductivity in the presence of gas molecules.
- *Mass-sensitive sensors*, such as quartz crystal microbalance (QCM) sensors [13] and surface acoustic wave (SAW) sensors [14], detect gas adsorption by measuring shifts in resonant frequency.
- *Electrochemical sensors* [15] convert chemical reactions at the electrode surface into electrical signals.
- *Optical sensors* [16] monitor changes in light absorption, fluorescence, or scattering in response to gas exposure.
- *Field-effect transistor (FET)-based sensors* [17,18], a growing category often involving nanomaterials or 2D materials, modulate current flow through a semiconductor channel when exposed to target VOCs.
- *Bioelectronic sensors* [19] integrate biological recognition elements to selectively detect specific VOCs.

Table 1 presents the advantages and application domains of the types of sensors that can be used in the e-nose development process.

The *signal processing unit* transforms sensor outputs into digital electronic signals for further analysis. It includes amplifiers, analog-to-digital converters (ADC), and noise filters to refine the data.

The *pattern recognition system* uses machine learning, artificial intelligence, or statistical algorithms to analyze the sensor data and identify unique odor patterns by comparing against a database of known smells. The most commonly used approaches include principal component analysis (PCA) [20] (reduces the dimensionality of sensor data while retaining key information), linear discriminant analysis (LDA) [21] (classifies odors by maximizing the separation between different groups), artificial neural networks (ANNs) [22] (mimics brain-like processing to learn and recognize odors patterns), support vector machines (SVMs) [23] (separate odor data into different classes using an optimal decision boundary), and k-nearest neighbors (KNNs) [24] (classify odors based on similarity to known reference samples). Within an e-nose, the sensor type determines the data characteristics, which in turn influence the pattern recognition technique needed for effective odor analysis. Therefore, MOS and CP sensors require fast, efficient recognition methods like PCA, ANNs, and SVMs due to large sensor response variations; QCM and SAW sensors produce high-precision frequency-based data, making ANNs and SVMs ideal for classification; optical and electrochemical sensors work well with PCA and LDA for chemical discrimination; and bioelectronic sensors use advanced techniques like ANNs to handle complex biological interactions in odor detection. Table 2 presents the advantages of each pattern recognition technique that can be used in the e-nose development process, the relation between these techniques and sensor types, and also the areas where they can be used.

We found that the results of the odor classification and analysis should be presented in a more user-friendly manner (i.e., mobile and web-based interfaces), making them easily accessible to e-nose end-users. This approach enhances efficient user interaction, enables real-time monitoring, and improves data visualization.

In addition to the previously discussed components, an e-nose should include a *sample delivery system* to ensure consistent, controlled, and repeatable exposure of VOCs to the sensor array. This component plays a key role in enhancing the accuracy and dependability of odor detection. It can include a sampling port to collect the gas sample, pre-concentrators to enhance sensitivity by capturing and releasing VOCs, filters to remove unwanted contaminants, flow controllers to regulate the gas rate and pressure, temperature and humidity controllers to maintain optimal conditions to prevent variations in sensor responses, a sample chamber to hold the gas sample for uniform interaction with the sensor array, and pumps to facilitate the movement of the gas sample through the system, and a ventilation system to ensure safe disposal of the analyzed gas after detection [25].

**Figure 1:**
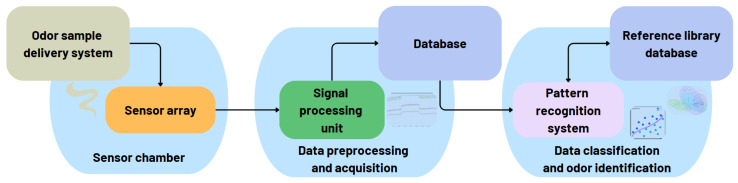
The main components of e-nose and their interactions.

Figure 1 presents an overview of the e-nose components, highlighting the key elements we have discussed above and their interconnections.

## 3. Review Methodology

### 3.1. Criteria for Selecting Relevant Research Papers Used in This Review

**Figure 2:**
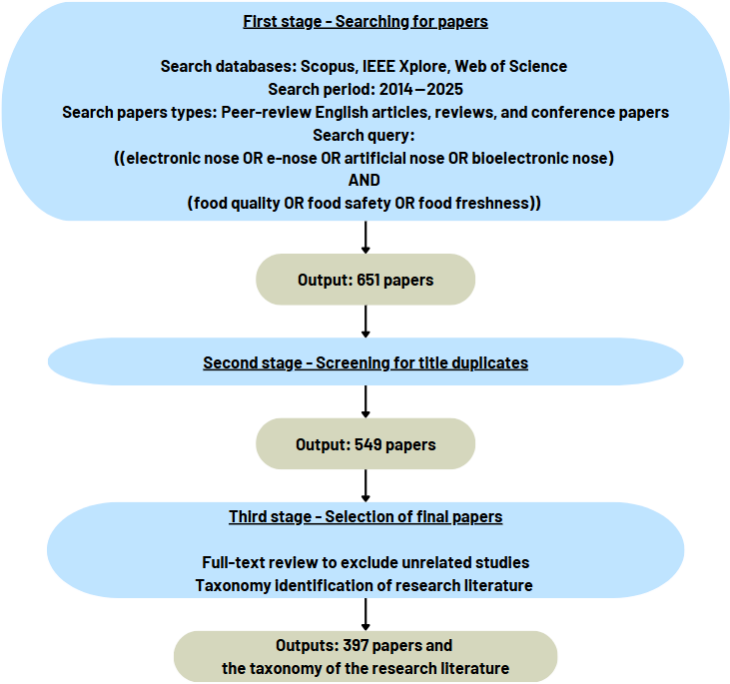
The three stages of the review methodology.

To select the most relevant research papers used in this review, we adopted the strategy in Figure 2.

1. In the first stage, we used the Scopus, IEEE Xplore, and Web of Science (WoS) electronic databases to search for final peer-review English-language documents published between 2014 and 2025. We considered only documents of type articles, reviews, and conference papers. We conducted the search using the following words appearing in the title or abstract of the documents: electronic nose, e-nose, artificial nose, bioelectronic nose, food quality, food safety, and food freshness.
2. In the second stage, we reviewed the titles of the documents retrieved from the search query to remove duplicates.
3. In the third stage, we thoroughly reviewed the full text of the remaining documents (after removing duplicates) before making the final selection, excluding unrelated studies, highlighting the key sections relevant to our review, and identifying the taxonomy of the research literature on the development of e-nose technology/applications in the field of food quality and safety.

### 3.2. Preliminary Results Obtained

The search query results from the Scopus electronic database, retrieved on 6 March 2025, yielded a total of 69 peer-reviewed documents. These include 40 journal articles (58%), 11 reviews (15.9%), and 18 conference papers (26.1%).

The search results from the IEEE Xplore database, retrieved on 6 March 2025, reveal a total of 117 peer-reviewed documents. These include 21 journal/magazine articles (18%), 2 reviews (1.7%), and 94 conference papers (80.3%).

The search results from the Web of Science database, retrieved on 6 March 2025, indicate a total of 465 peer-reviewed documents. These include 298 journal articles (64.1%), 120 reviews (25.8%), and 47 conference papers (10.1%).

After removing the duplicates, the preliminary documents dataset consists of 314 journal/magazine articles, 122 reviews, and 113 conference papers.

Table 3 presents an overview of the preliminary search results for the documents.

### 3.3. Final List of Selected Papers and Taxonomy of the Research Literature on E-Nose for Food Quality and Safety

After a comprehensive analysis of the publications obtained in the second stage of the selection process, we excluded those that (a) do not have food quality and safety as their main research objectives (with terms like food, food quality, food safety, or food freshness appearing only in the abstract); (b) primarily review the food quality and safety field, presenting only general information about e-nose, such as definitions and descriptions of basic components, alongside other systems, devices, or techniques for this purpose; (c) rely only on the gas chromatography technique; (d) address food quality for animals; and (e) do not show promising results in terms of performance metrics.

As a result, the final list of publications includes 397 documents, which include 241 journal/magazine articles, 80 reviews from journals and conferences, and 76 conference papers from all the three electronic databases previously mentioned. Table 4 presents a summary of the final list of publications.

**Figure 3:**
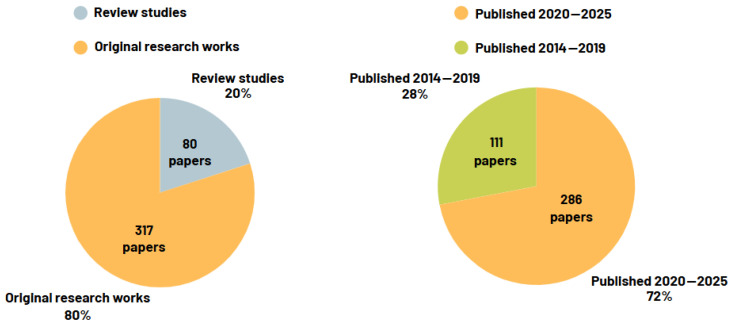
Statistics from the final list of publications.

Figure 3 presents an additional analysis of the data, which reveals that approximately 20% of the papers in the final dataset are review studies, and most of the original research results have been published in journals/magazines. Additionally, 72% of the papers were published in the past 5 years, which demonstrates the growing interest of the scientific community in this topic.

**Figure 4:**
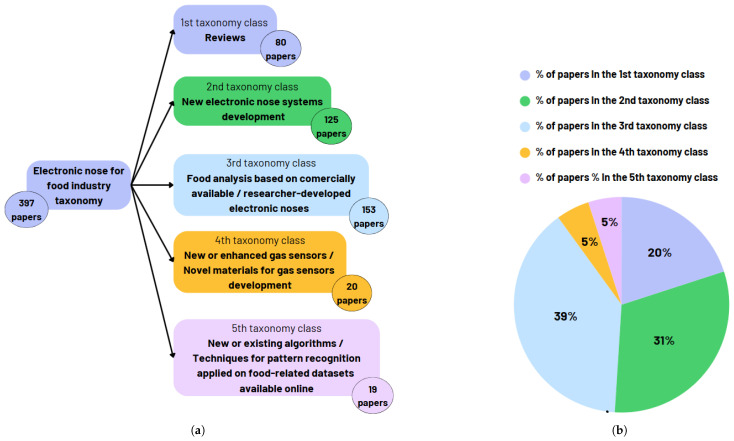
(**a**) Taxonomy of the research literature on electronic nose for food quality and safety. (**b**) The distribution (by categories) of papers in the final list of publications.

During this stage, we classified the documents in the final dataset into five distinct classes. The first category covers review studies that focus on the application of e-nose technology in the food industry. The second category includes papers that present the development of new electronic noses for food quality and safety. The third category comprises papers that use commercially available or researcher-developed electronic noses, either alone or in combination with other techniques, for food analysis. The fourth category includes research studies that introduce new/enhanced gas sensors or novel materials for gas sensor development with applicability in the food industry. The last category includes papers that present the outcomes of applying existing algorithms or techniques for pattern recognition or their fusion, on food-related datasets available online. Figure 4a illustrates the taxonomy of the research literature on e-nose technology for food quality and safety based on these categories. Figure 4b presents the distribution of papers in the final list of publications belonging to each identified category.

As Figure 4 shows, most of research efforts have focused on analyzing food data from commercial or laboratory-based e-nose systems, followed closely by studies dedicated to developing new electronic nose systems. There are also several reviews that have been published that cover the latest advancements in the field. Studies concerning the development of new gas sensors and new algorithms/techniques for pattern recognition using existing datasets have also attracted some interest.

## 4. Analysis of the Research Works from the Final List of Selected Publications

In this section, we emphasize the novel contribution of our review when compared with existing reviews that we selected in our taxonomy. Additionally, we present the most promising research works from the final selection, encompassing the remaining four categories of the taxonomy, and we highlight the key lessons learned from these past studies.

### 4.1. Reviews

As Figure 4 shows, we identified several review articles in our final selection of papers. These published reviews covered several aspects on the topic of electronic nose.

#### 4.1.1. Reviews on Advancements in Electronic Nose Systems for Food Industry Applications

One category of review papers explored recent advancements in electronic nose systems and their applications in the food industry. The studies [26,27,28,29,30,31,32,33,34,35,36,37] describe the technologies used to develop each of the e-nose components and discuss some proposed solutions of this type of systems. Some reviews focus on the advancement of electronic nose technology in relation to a specific food category: meat [38,39,40,41], berries [42], oils [43,44], fruits and vegetables juice [45], milk and dairy [46,47], tea [48], and wine [49,50].

#### 4.1.2. Reviews on Sensor Development and Pattern Recognition Techniques in Electronic Nose Systems

Reviews such as [51,52,53,54,55,56,57,58] investigate the progress made in the development of sensors with high sensitivity and selectivity for detecting VOCs emitted from food, and highlight the challenges related to sensor stability, cross-sensitivity, environmental interference, and also their integration into e-nose-based applications. The authors of [59,60,61,62] reviewed pattern recognition techniques that can be applied to electronic nose systems. In addition, they also discussed current challenges and potential future directions of these methods.

#### 4.1.3. Reviews on Recent Sensing Technologies for Food Quality Assessment

Another category of reviews focuses on recent sensing technologies for food quality assessment. For example, refs. [63,64,65,66,67,68,69,70,71,72,73] present comprehensive reviews of electronic sensing technologies (e-nose, e-tongue, e-eye) and their applications. Similarly, refs. [74,75] highlight electronic noses as intelligent detection tools in the food industry, along with technologies such as computer vision, intelligent tracing systems, intelligent colorimetric films, and near-infrared spectroscopy.

#### 4.1.4. Our Review on Sensor-Based Electronic Nose for Food Quality and Safety

Our review offers a comprehensive and structured examination of the past decade’s research on sensor-based e-nose devices for ensuring food quality and safety. In contrast with earlier efforts and past publications mentioned in Section 4.1.2, which focus on the technologies used to develop e-nose components and explore proposed solutions, the novelty of our contributions includes the following:

- *Development and application of a unique taxonomy ensuring broad coverage and reduced selection bias:* We conducted an extensive analysis of peer-reviewed studies across three major scientific databases, the taxonomy. The taxonomy enables a systematic evaluation of technological advancements (both sensors and pattern recognition techniques), practical implementations, and performance outcomes across the following food and beverage sectors: meat, seafood, vegetables and fruits, spices, oils, coffee, tea, diary, and alcoholic beverages.
- *Decade-long coverage of research results:* By capturing trends over an extended period, our review offers an up-to-date perspective on technological evolution and trends.
- *Lessons-learned synthesis:* Our review identifies critical lessons learned from the existing literature in each category from the taxonomy we developed. The lessons learned will help guide both future academic research and practical development of e-nose systems for food quality and safety.
- *Identification of unresolved research gaps:* Our review reveals notable gaps that must be addressed in the future. These gaps include the lack of e-nose real-world validation, limitations in sensor sensitivity and stability, challenges in achieving miniaturize and portable e-noses, lack of standardized testing protocols, limited real-time processing capabilities, and insufficient support for user-friendly visualization of odor classification and identification outcomes.

Our review serves as a valuable resource for researchers, especially those new to the field of electronic noses for food quality and safety, because it provides a comprehensive foundation and state-of-the-art, in-depth information on current technologies, applications, and research directions in this area.

### 4.2. Electronic Nose Systems for Food Quality and Safety

Thirty-one percent of the journal/magazine articles and conference papers in the final list of selected publications present electronic nose systems for odor authentication and recognition, for quality assessment, and for quality monitoring of various food and beverage products, including meat (i.e., chicken, beef, pork), seafood (i.e., fish, prawn), vegetables and fruits (i.e., tomato, broccoli, banana, avocado), spices, oils (i.e., palm, olive, sunflower, essential oils), coffee, tea, diary (i.e., milk), and alcoholic beverages (i.e., rice wine, beer, scotch, whiskey, liquor). These papers describe the physical components of the e-noses developed, along with the data analysis approaches used for odor classification and identification. Table 5 briefly presents the analysis of these e-noses from the following perspectives: design architecture (sensor array and signal processing unit), data analysis techniques, evaluation and performance metrics, and application area.

#### Lessons Learned

Based on the detailed analysis of electronic nose-type systems for food quality and safety that we performed above in Table 5, we found that most of these systems are homemade, low-cost, using commercial MOS sensors and simple microcontroller boards in the Arduino and ESP32 range (Arduino Uno R3, Arduino Mega 2560, Arduino Uno, ATmega8, Arduino Due, PIC, PIC18F45K22, Arduino Nano, Gizduino, ESP32, and the Node MCU IoT platform) or single-board computers such as Raspberry Pi (Raspberry Pi 3 and 4, S3C6410-based Linux platform), or data acquisition cards like NI DAQ card—USB-6009 and PCI6035E, available on the market. Table 6 provides a complete list of all the gas sensors used in e-nose solutions from Table 5 along with their key characteristics. The characteristics presented are important because sensor range defines e-nose sensitivity, and power and response time are critical for portable or multi-sensor systems. For example, heater-based sensors need long startup or preheat times, which limits portability. Table 5 and Table 6 offer researchers a comprehensive overview of the most commonly used sensors in the development of the e-nose system, their corresponding application areas, and the algorithms that achieve good performance levels. These tables provide practical insights and recommendations that will significantly reduce the efforts of designers and implementors required for sensor selection. Creating such selection tables is a crucial preliminary step in the sensor selection process.

For the classification of the acquired data, Table 5 reveals that the main techniques presented in Table 2 (PCA, LDA, ANNs, SVM, KNN) and variations of those (i.e., CNN, PNN, LSTM, MLP, SVR) are the most used. Current trends focus on implementing machine learning technologies, such as CNN and LSTM, and general AI solutions, because processing platforms have become more accessible, affordable, and powerful. Although these solutions involving AI methods and algorithms are not novel, researchers can now truly benefit from their use, thanks to the emergence of cost-effective and high-performance hardware platforms. The use of other models, such as Linear Regression, k-Means, Random Forest, Stochastic Gradient Descent, Naïve Bayes, Fuzzy logic, Discrete Fourier Transformation, Quadratic Discriminant Analysis, and Extreme Gradient Boosting, also yielded results with an accuracy of over 80%, as reported by the authors. Some research works combined PCA with other techniques (i.e., SVN, LDA, PNN, KNN, k-Means) for the following reasons: (a) it reduces the number of features of the acquired data while retaining the most relevant information; (b) it helps filter out sensor noise and irrelevant variations in the data; (c) it pre-processes the data, which results in faster training times, better generalization, higher accuracy, especially when the raw sensor data are noisy or redundant; and (d) it reduces data to two or three dimensions, guiding researchers in the selection of the appropriate pattern recognition technique. In these cases, the performance metrics yield highly encouraging results. We can conclude that PCA acts as a smart pre-processing step that makes the data more manageable and informative for learning tasks.

Most proposed e-nose solutions lack support for the user-friendly visualization of the odor classification and the identification of the results. It is important to ensure that the analysis results are effectively communicated to end users in real time through software applications and easy-to-use graphical user interfaces.

Our in-depth analysis of the research studies also reveals that the developed e-nose systems do not target the achievement of high-performance measurement prototypes or products; rather, the authors focused on concept validation with their prototype systems. This is supported by the following conclusions:

- *Cost:* The sensors used in the experiments belong to the cheap components’ class, usually included in gas measurement systems, where their main feature is the detection of the presence of a certain gas component. Another characteristic of such systems is the low manufacturing cost. The documentation that comes with the sensors used is brief, containing little relevant information, omitting aspects like the manufacturer-recommended schematics, calibration and compensation methods depending on temperature and relative humidity values, or formulas for converting the voltage or resistance measured by the microcontroller back into the actual physical quantity measured by the sensor. In many cases, the datasheets do not include important characteristics such as precision, accuracy, repeatability, stability over time, or startup periods. Most sensors used are analog, and they do not integrate calibration circuits, drift, compensation or control mechanisms, or an ADC within the same package. As a result, their overall measurement performance is typically poor, and they are further affected by the required external electronics. The BME688 [125] sensor used in [124,152] stands out in a positive way because it includes important circuitry besides the sensing element, which supports advanced functions such as filtering, signal conditioning, the ADC, the compensation table and algorithm, and digital communication with the processing unit, ESP32. The BME688 development kit uses eight sensors instead of one to form a sensor array, which enhances detection performance, especially for low-cost setups. Though calibrated, sensors differ slightly, and tracking signal trends over time across multiple sensors improve reliability. Additionally, free gas flow causes variations in individual sensor responses before steady state, making arrays beneficial.
- *Power usage:* The energy consumption required by the sensors used is high, and they are suitable for integration with systems powered permanently from the main power outlet. Sensors with heaters that are common in most studies have long response times, between 10 and 300 s, and operate at 200–400 °C. This leads to high power consumption unsuitable for portable devices and faster aging that requires frequent recalibrations. The recommended preheating time, or sensor warm-up time, until the first correct measurements can be extensively long, up to 2 to 7 days in some cases. The power consumed during measurement ranges from 0.3 to 1.0 W, and the continuous operation of the heating element in some sensors makes them unsuitable for use in portable electronic nose systems.
- *Data collection:* The data acquisition platforms are not designed for instrumentation systems. Most of the proposed solutions use low-resolution ADCs (10- or 12-bit), typically with a 0 to 5 V input range. This leads to an effective resolution per bit of 5 to 10 mV. Temperature and relative humidity compensation are generally based on low-accuracy T and RH sensors (±1 °C for temperature and ±4% for relative humidity), with a few exceptions. In platforms based on ESP32, measurement performance in terms of used digits is further limited by the built-in ADC, which typically offers an effective resolution of only 8 bits. Some proposed electronic nose systems utilize industrial-grade measurement platforms (i.e., PCI6035E, AD7606) and compensation sensors (sensors helping in adjusting the measurement depending on ambient parameters such as temperature, humidity, or pressure) for temperature and relative humidity (i.e., SHT15), which outperform those commonly used in standard gas detectors.
- *Portability:* Very few papers focus on low-power or portable systems. However, the question of whether a measurement system can be powered from the main outlet is a valid one. In this case, what are the time and logistical efforts required to make the system operational at a different location? For example, when considering the BME688 sensor, a system using it requires 30 min to reach maximum accuracy after power cycling. Other manufacturers do not specify this time requirement, but in some cases, it could require days.
- *Data processing:* Generally, the accuracy of measurement data is verified through thousands of hours of operation and repeated measurements, ideally conducted on multiple similar devices operating in parallel. Some past research solutions [103,115,119,138,141,163] used professional techniques (i.e., Gas Chromatography–Mass Spectrometry (GC–MS) [176]) to compare their experimental results with reference ones. In all the papers that we reviewed, the number of samples collected by the sensor arrays and used by the machine learning algorithms is rather small (<300 samples). In these conditions, expecting authors to validate their work with equipment that has been running for a full year is not feasible. This raises questions regarding the performance metrics obtained. To validate the results obtained, standard test/evaluation scenarios should be run, not just particular test sets created by the authors of the papers.

### 4.3. Food Analysis Based on Previously Developed/Commercial Electronic Nose Systems

Thirty-nine percent of the publications in the final set of selected publications present food and beverage quality assessment and quality monitoring solutions that integrate commercial e-noses or electronic nose systems developed by other research labs. The research works in this taxonomy category perform some sort data acquisition based on these devices, followed by, in the majority of cases, different data processing and prediction algorithms.

#### 4.3.1. Commercial Electronic Nose Systems

Our analysis found that the most used commercial e-noses are PEN3 [177] from Airsense Analytics Inc., Fox 3000 [178] from Alpha MOS, Fox 4000 [179] from Alpha MOS, FOODsniffer [180], NeOse Pro [181] from Aryballe Technologies, and Cyranose^@^ 320 [182] produced by Sensigent. Table 7 summarizes their main characteristics, along with a few promising papers that employed them, considering aspects such as data analysis techniques, evaluation and performance metrics, and application domains.

#### 4.3.2. Electronic Noses Developed by Academic Research Groups

Our analysis reveals that several academic research groups developed electronic noses that other researchers later employed them in their own studies. For instance, research groups from the University of Rome Tor Vergata developed LibraNose [203] and also other prototypes [204,205], very similar to LibraNose. Researchers from the Industrial Engineering School of the University of Extremadura, Spain, designed a low-cost, high-accurate electronic nose [206], and those from the Institute of Agrophysics PAS in Lublin, Poland, created the Agrinose [207] system. In the Department of Biosystems Engineering, Faculty of Agriculture, Bu-Ali Sina University, Hamedan, Iran, researchers designed an e-nose [208] that has been successfully used in several studies regarding garlic quality assessment. Table 8 summarizes the main characteristics of these e-noses, along with a few promising papers that employed them, considering aspects such as data analysis techniques, evaluation and performance metrics, and application domains.

#### Lessons Learned

Numerous research studies use commercial electronic nose instruments, like those mentioned in Table 7, because of their user-friendly operation and dependable performance. These instruments allow researchers to save time, ensure consistent reliability, and focus on more advanced research questions. Additionally, commercial e-noses facilitate the generation of reproducible and comparable results, all without the need to navigate the challenges of developing custom hardware.

Our analysis highlights several ways in which the research community adopted commercial electronic noses, as follows:

- *To apply well-known pattern recognition techniques to assess the quality and safety of different types of food:* Studies such as [178,188,194,195,196] used PEN3, Fox 3000, and Fox 4000 commercial electronic noses to apply algorithms such as PCA, Random Forest, and ANNs to evaluate the quality of various food categories (meat, fruits, jams, milk). In [186,189,197], the researchers applied well-known pattern recognition techniques (PCA, PLS-DA, Partial Least Squares regression) to both e-nose data (PEN3, Fox 4000) and chromatography analysis results (GC–MS), leveraging the complementary strengths of these methods in chemical analysis and pattern recognition.
- *To develop novel models or algorithms for odor identification and classification:* Studies such as [183,191,193,202] used data collected from commercial electronic nose devices (PEN3, Cyranose^@^ 320) to develop novel data models for the identification and classification of odors, demonstrating strong performance.
- *To confirm the ability of commercial e-noses to recognize and classify aromas:* Studies such as [199,200] proved that the FOODsniffer e-nose can accurately classify meat based on its data analysis, with results validated against GC–MS analysis and physicochemical measurements. The authors of [201] validated NeOse Pro to evaluate the quality of the plant-based beverage by applying PCA and LDA to the e-nose data and comparing the results with those obtained using the same algorithms on GC–MS data.

The previous classification also applies to electronic noses developed by research groups. Table 8 shows that the e-nose designed within the Department of Biosystems Engineering, Bu-Ali Sina University, can successfully assess the quality of garlic by applying well-known pattern recognition techniques (PCA, LDA, SMV, Backpropagation Neural Network) [224], and the e-noses developed by the University of Rome Tor Vergata work well with PLS-DA, PCA, and LDA to identify bacteria in food and beverage [204,205,213]. In [206,209,218], researchers applied techniques such as PCA, Random Forest regression, PLS-DA, and Partial Least Squares to data collected from LibraNose and the e-nose developed by the Industrial Engineering School of the University of Extremadura to assess the quality of meat, roasted coffee, and almonds. The authors of these studies validated the obtained results using GC–MS and High Performance Liquid Chromatography. Studies [210,211,212] used LibraNose data in the development of novel models or algorithms for meat quality assessment, while other studies [221,222] used Agrinose to implement models for the assessment of the suitability of bread for consumption after storage and to identify rapeseed spoilage. These last two studies validated their results by comparing them with those obtained from GC–MS and Fourier Transform Infrared Spectroscopy analysis.

During our analysis, we identified new sensors integrated into e-noses developed by research groups. Table 9 completes the list of sensors presented in Table 6 by including the new ones mentioned in Section 4.3.2 of this review.

An in-depth analysis of the research studies reveals that these instruments can accurately detect certain types of food, but this does not necessarily imply they are suitable for detecting all food classes. The selection of such instruments should not be arbitrary; the selected sensors must be carefully evaluated based on the primary VOCs present in the target sample. Comparing the results obtained from using e-nose data and various algorithms with those from chromatography analyses, which are highly accurate in the identification of VOCs in food, serves to validate the findings. Chromatography provides precise, specific chemical data, but is time-consuming, expensive, and requires skilled operators. E-noses may be more advantageous over GC–MS in distinguishing the integral aroma profile, although they cannot identify the explicit VOCs of different samples. Combining e-noses data analysis with complementary technologies analysis, such as human sensory evaluation, GC–MS, or e-tongue to assess food quality can achieve high detection accuracy. However, this approach often involves significant time due to data fusion processes and incurs substantial costs.

Similar to the approaches analyzed in the first category of our taxonomy, those in the second category also focus on concept validation conducted at the laboratory level.

### 4.4. Gas Sensors for Electronic Nose Systems

In the final set of selected publications, we also found several research efforts that focus on the development of new gas sensors for the food industry. Table 10 summarizes the key results from the selected articles.

#### Lessons Learned

Recent advancements in e-nose technologies demonstrate that sensor performance can be significantly improved through material innovation and system integration.

Surface and chemical modifications, such as those applied to Silicon NanoWires or graphene, enhance sensitivity and selectivity, which is critical for detecting specific analyses in complex environments. The use of advanced materials like graphene and graphene with Metal Phthalocyanines enables precise gas discrimination, expanding the applicability of e-noses in food quality and safety.

Bio-inspired approaches, particularly those mimicking the diversity of biological olfactory systems, show strong potential when combined with AI and machine learning for analyzing multi-dimensional VOCs data. Moreover, the integration of e-nose systems with compact, low-power computing architectures, such as memristor-based accelerators, addresses challenges in energy efficiency and real-time data processing, essential for portable devices.

Efforts toward miniaturization, including the use of Film Bulk Acoustic Resonator sensors and reference drift compensation, have made portable e-noses more viable for field deployment. Colorimetric and bioelectronic sensor innovations, leveraging either chemical complexes or olfactory receptors, offer energy-independent or ultra-sensitive detection, pushing the boundaries of low-resource and high-precision sensing.

These developments show that future e-nose systems will increasingly rely on the synergy of novel materials, bio-inspired sensing strategies, smart signal processing, and system-level integration to meet the demands of next-generation applications in food quality and safety assessment.

### 4.5. Food-Related Datasets and Algorithms/Techniques for Pattern Recognition Used on Them

Several publications in the final collection of articles emphasize the use of available food-related databases, which various algorithms/techniques for pattern recognition used. Table 11 summarizes the e-nose datasets and the related studies that utilized them, as well as the methods and performance metrics reported.

#### Lesson Learned

Our analysis reveals the following results:

- Public datasets provide a valuable foundation for developing and testing new models or algorithms for odor identification and classification.
- Public datasets accelerate comparative research. The availability of well-structured datasets has enabled researchers to benchmark different models, promoting transparency and repeatability. As Table 11 shows, deep learning models outperform traditional classifiers in some cases. Additionally, approaches that combine multiple classifiers tend to boost accuracy and model stability. Such comparisons are possible because the researchers employed the same dataset.
- Diverse model strategies provide complementary insights. The use of a wide range of algorithms across datasets shows that no single approach performs best across all datasets and applications. Different algorithms excel under specific data characteristics and task requirements.
- Model performance is dataset dependent. Even if the authors of the cited research efforts reported high accuracies, these are heavily influenced by the specific dataset, number of classes, sensor types, and experimental conditions.

## 5. Research Gaps and Future Research Opportunities

Despite promising progress, a significant gap remains between lab scale e-nose prototypes and practical, market-ready solutions for food quality and safety monitoring. Current limitations include insufficient sensor sensitivity and stability, challenges in device miniaturization and portability, lack of standardized testing protocols, limited real-time processing capabilities, and lack of support for the user-friendly visualization of the odor classification and identification results. Furthermore, there is a lack of robust data fusion strategies and comprehensive odor reference datasets to support reliable decision making in diverse real-world scenarios.

To address these gaps, future research should focus on the following areas:

- *Sensor technology:* We must develop novel gas-sensitive materials with enhanced selectivity and sensitivity for food VOCs, new gas sensors with fast response time, adaptive calibration methods, and sensor baseline correction techniques to improve the stability of the gas sensors and integrate bio-inspired or biomimetic sensors.
- *Data processing:* We must implement deep learning algorithms for pattern recognition and VOC classification. We must develop and implement efficient multi-sensor (e-nose, e-tongue, e-eye) data fusion algorithms for a more holistic food profiling approach. Additionally, we must also develop standardized odor databases and reference libraries and real-time data analysis platforms for on-site decision making.
- *Miniaturization and portability:* We must integrate micro-electro-mechanical systems/ nano-electro-mechanical systems technology for compact and low-power devices. We must also develop reliable wireless and IoT-enabled e-noses for remote monitoring.
- *Standardization:* We must develop standardized testing protocols across different food types and storage conditions.

To consolidate the practical contributions of this review, Table 12 summarizes the key research gaps identified in current electronic nose applications and the corresponding solutions proposed in this work.

## 6. Conclusions

The application of electronic noses in the food industry has witnessed significant growth over the past decade, demonstrating considerable potential in monitoring food quality and safety, detecting spoilage, assessing freshness, and verifying food authenticity across a wide range of products, including meat, seafood, fruits and vegetables, spices, oils, dairy, and beverages. We arrive at this conclusion based on our analysis of more than 350 peer-reviewed documents retrieved from three scientific databases (Scopus, IEEE Xplore, WoS) using a targeted keyword search. This analysis followed a proposed taxonomy that categorized the publications into five distinct classes: (1) publications that present the development of new electronic noses; (2) publications that use commercially available or researcher-developed electronic noses, either alone or in combination with other techniques; (3) publications that introduce new/enhanced gas sensors or novel materials for the development of gas sensors; (4) publications that present the outcomes of applying existing algorithms or techniques for pattern recognition, or their fusion, on food-related datasets available online; and (5) review studies. Moreover, the analysis revealed that, despite notable progress, several challenges remain. E-nose systems still rely on general-purpose sensor arrays, limiting their adaptability to diverse types of food. In addition, issues related to sensor noise, drift, calibration, temperature, or modularization continue to hinder widespread industrial adoption. Moreover, while many studies report high classification accuracy, there is often a lack of standardization in methodologies/protocols for data pre-processing, feature selection, model deployment, and testing, along with limited application in real-world scenarios. Future research directions must focus on gas sensor technology, data processing, miniaturization and portability, and standardization.

## Figures and Tables

**Table 1:** E-nose sensor array types, their advantages, and application sectors.

Type of Sensor	Advantages	Application Sector
Chemiresistive sensors: MOS	High sensitivity, high selectivity, durability, long lifespan, fast response time	Air quality monitoring, food freshness detection, industrial gas sensing, medical diagnostic
Chemiresistive sensors: CNT	Ultra-high sensitivity, fast response time, low power consumption, miniaturization potential	Breath analysis for disease detection, air quality monitoring, workspace safety
Conductometric sensors: CP	Fast response time, low power consumption, tunable sensitivity	Medical diagnostics, food quality assessment, environmental monitoring
Mass-sensitive sensors: QCM	High sensitivity, ability to detect low-concentration gases	Breath analysis, detection of toxic substances, fragrance quality control
Mass-sensitive sensors: SAW	Fast response time, small size, high ruggedness	Explosive and drug detection, environmental monitoring, workspace safety
Electrochemical sensors	High selectivity in terms of the electrochemical properties of target VOCs, low power consumption, reliable detection of specific gases	Toxic gas detection, breath analysis, air quality monitoring
Optical sensors	Non-contact sensing, high specificity in terms of the chemical identity of VOCs, fast response time	Industrial gas detection, medical diagnostics, hazardous material monitoring, food quality assessment
FET sensors	High sensitivity, fast response time, compatibility with nanomaterials and 2D materials, fast electronic response	Medical diagnostics, food quality assessment, environmental monitoring, industrial process control, security and defense
Bioelectronic sensors	High specificity in terms of molecular recognition of target VOCs, biomimetic functionality, potential for personalized diagnostics	Disease detection, food quality monitoring

**Table 2:** Pattern recognition technique, their advantages, related sensor type, and application areas.

Pattern Recognition Technique	Advantages	Type of Sensor	Application Sector
PCA	- Reduces data dimensionality without losing key information - Enhances visualization and interpretation - Improves classification performance - Speeds up computational processing - Enables unsupervised odor classification	MOS, CP, QCM, SAW, CNT, optical sensors, electrochemical sensors	Food quality control, medical diagnostics, environmental monitoring, industrial process control
LDA	- Maximizes odor class separation - Enhances classification accuracy - Reduces data dimensionality - Speeds up computational processing - Suitable for small and well-defined datasets	MOS, CP, QCM, SAW, optical sensors, electrochemical sensors	Medical diagnostics, food quality control, environmental monitoring, security and defense
ANNs	- Can handle nonlinear and complex data - High accuracy in odor classification - Self-learning and adaptability - Real-time processing capability - Multi-modal data fusion - Noise tolerance	MOS, CP, QCM, SAW, CNT, bioelectronic sensors	Medical diagnostics, food quality control, environmental monitoring, security and defense
SVM	- High classification accuracy - Handles nonlinear and high-dimensional data - Effective for small datasets - Works well with multiple sensor types - Suitable for binary and multi-class odor classification - Works well in real-time applications	MOS, CP, QCM, SAW, CNT	Medical diagnostics, food quality control, environmental monitoring, security and defense, workspace safety
KNN	- Easy to implement and interpret - Effective for small and medium-sized datasets - Works well with multiple sensor types - Adaptable for classification and regression - Suitable for real-time odor analysis	MOS, CP, QCM, SAW, CNT	Food quality monitoring, environmental monitoring, medical diagnostics, industrial process control

**Table 3:** Preliminary search results.

6 March 2025	Journal/ Magazine Articles	Reviews	Conference Papers	Total
Scopus	40	11	18	**69**
IEEE Xplore	21	2	94	**117**
WoS	298	120	47	**465**
**Total**	**359**	**133**	**159**	**651**
**Total without duplicates**	**314**	**122**	**113**	**549**

**Table 4:** Final search results.

6 March 2025	Journal/ Magazine Articles	Reviews	Conference Papers	Total
Scopus	3	2	6	**11**
IEEE Xplore	5	0	35	**40**
WoS	233	78	35	**346**
**Final total**	**241**	**80**	**76**	**397**

**Table 5:** Summary of electronic nose systems for food quality and safety.

Paper	Design Architecture	Data Analysis Techniques	Evaluation and Performance Metrics	Application Area
**Electronic Nose Systems for Meat Quality and Safety**				
[76]	Sensor array: MQ-137, MQ-136 [77], TGS2602 [78]; Signal processing unit: Arduino Uno R3	PCA	Accuracy: 94.9% (fresh/spoiled/rotten)	Beef quality assessment
		PCA + Probabilistic Neural Network (PNN) [79]	Accuracy: 100% (fresh/spoiled)	
[80]	IoT-enabled e-nose; Sensor array: AM2302 [81], one optical sensor from Winsen Electronics Technology Co., Zhengzhou, China, MH-Z19C [82], ZE03-NH3, ZE03-C2H4 [83]; Signal processing unit: ESP32-S3 controller [84]	Linear Regression [85]	Aerobic bacteria and Pseudomonas species play a crucial role in the production of VOCs in beef	Beef quality monitoring and spoilage detection
[86]	Sensor array: MQ-2, MQ-3, MQ-4, MQ-6, MQ-7, MQ-8, MQ-9, MQ-135 [77]; Signal processing unit: Arduino Mega 2560 microcontroller [87], Raspberry Pi 4 [88]	PCA + SVM	Accuracy: 98.49% (healthy/ compromised)	Chicken meat quality assessment
[89]	Sensor array: MQ-2, MQ-3, MQ-6, MQ-7, MQ-9, MQ-135, DHT22 [90]; Signal processing unit: Arduino Uno microcontroller	SVM, Linear Regression, KNN, Random Forest [91]	Best accuracy: 100% for Random Forest with random split data; 69% for Random Forest with non-randomly split data; 78.5% for SVM with group split data (fresh/semi-fresh/spoiled)	Chicken meat quality assessment
[92]	Sensor array: HGS1000, HGS1001, HGS1002, HGS1007 [93]; Signal processing unit: 12-bit ADC with four channels of input data; heating voltage can be set between 0 and 2.4V	Convolutional Neural Network (CNN) CNN [94] + time series feature extraction [95]	Accuracy: 92.1% (fresh/sub-fresh/spoiled) Accuracy: 98.4% (fresh/sub-fresh/spoiled)	Pork, beef, mutton, chicken, crab, shrimp, fish meat quality assessment
[96]	Sensor array: MQ-2, MQ-4, MQ-6, MQ-9, MQ-135, MQ-136, MQ-137, MQ-138,DHT22; Signal processingunit: N/A	KNN	Accuracy rates between 97% and 100% (variations of meat with ratio 0%, 10%, 50%, 90%, 100%)	Authenticity of beef and pork meat
		SVM	Accuracy rates between 81.5% and 99.5% (variations of meat with ratio 0%, 10%, 50%, 90%, 100%)	
**Electronic Nose Systems for Seafood Quality and Safety**				
[97]	Sensor array: MQ-136, MQ-137, MQ-5, MQ-8; Signal processing unit: N/A	Support Vector Machine Regression Technique (SVR) [98]	R-squared (R^2^): 0.981; Root Mean Square Error (RMSE): 0.012	Estimation of the microbial population in seafood
[99]	IoT-enabled e-nose with image processing capabilities; Sensor array: N/A; Signal processing unit: N/A	A nonparametric kernel-based modeling + hidden Markov model	Quality model indices closely align with the manual results provided by quality assurance experts	Fish origin verification, fish quality assessment
[100]	Sensor array: MQ-1, MQ-2 and two MQ-135; Signal processing unit: ESP32 microcontroller	KNN	Accuracy: 98% (fresh/less fresh/ not fresh)	Freshness and quality of crabs
		Naïve Bayes [101]	Accuracy: 91% (fresh/less fresh/ not fresh)	
		SVM	Accuracy: 87% for SVM (fresh/less fresh/not fresh)	
[102]	Sensor array: TGS2620, TGS2611, TGS822, TGS832, TGS2602, TGS2600, TGS826, TGS825; Signal processing unit: N/A; Preheating process before using the sensors	PCA	Cumulative variance of the principal component: 95% (fresh/contaminated)	Tuna quality assessment (*Pseudomonas aeruginosa* bacteria)
		SVM	Accuracy: 99% (fresh/contaminated)	
[103]	Sensor array: MQ-2, MQ-3, MQ-4, MQ-5, MQ-6, MQ-7, MQ-8, MQ-9; Signal processing unit: N/A	Linear Regression	R^2^: 0.98; Accuracy: 93.75%	Prawn quality assessment
**Electronic Nose Systems for Vegetables and Fruits Quality and Safety**				
[104,105]	Sensor array: MQ-3, MQ-6, MQ-8, MQ-135; Signal processing unit: ADC for Raspberry Pi 4/ Raspberry Pi 3	CNN	Accuracy: 86% (ripe/not ripe/unknown)	Identification of the ripening stage of tomato fruits
[106]	Sensor array: MQ-135, MQ-136, TGS822, TGS2600, TGS2602, TGS2603, TGS2610, TGS2611, DHT22; Signal processingunit: N/A	Random Forest	Accuracy: 94% (good/good/fair/ poor)	Identification of the ripening stage of tomato fruits
		KNN	Accuracy: 83% (good/good/fair/ poor)	
		ANNs	Accuracy: 79% (good/good/fair/ poor)	
		SVM	Accuracy: 64% (good/good/fair/ poor)	
[107,108]	Sensor array: MQ-135, MQ-4; Signal processing unit: Gizduino micro- controller [109]	ANNs	Accuracy: 93.33% (not spoiled/ partially spoiled/ spoiled)	Tomato puree quality assessment
		Fuzzy logic technique [110]	Accuracy: 90% (not spoiled/ partially spoiled/ spoiled)	
[111]	Sensor array: TGS2620, TGS823, DHT22; Signal processing unit: Arduino microcontroller	PCA + t-distributed Stochastic Neighbor Embedding [112] + k-Means [113] + Long Short-Term Model [114]	Accuracy: 99.46% (fresh/half-spoiled/spoiled)	Broccoli quality assessment
[115]	Sensor array: TGS880, TGS822, TGS826, TGS2602, TGS2600; Signal processing unit: ATmega8 [116] microcontroller with an integrated ADC	PCA + Centroid link- and completely-link cluster analyses	Similarity levels >93% for 3/4 of the samples tested (fresh/half/ completely contaminated)	Broccoli quality assessment (Staphylococcus, Salmonella and Shigella)
[117]	Sensor array: MQ-2, DHT11; Signal processing unit: Arduino Uno microcontroller and Node MCU [118] IoT platform	Linear Regression, Random Forest, SVR	Best performance with a value of Mean Squared Error (MSE): 0.1207 for Random Forest	Banana freshness assessment
[119]	Sensor array: TGS2600, TGS2602,TGS2603, TGS2610, TGS2611, TGS2612, TGS2620; Signal processing unit: NI DAQ card, USB-6009 [120]	PCA + KNN	Accuracy: 98.10% (unripe/half-ripe/fully ripe/overripe)	Identification of the ripening stage of banana
		PCA + SVM	Accuracy: 95.24% (unripe/half-ripe/fully ripe/overripe)	
		LDA + KNN	Accuracy: 90.48% (unripe/half-ripe/fully ripe/overripe)	
		LDA + SVM	Accuracy: 86.67% (unripe/half-ripe/fully ripe/overripe)	
[121]	Sensor array: MQ-2, MQ-3, MQ-4, MQ-5, MQ-7, MQ-8, MQ-135 sensors; Signal processing unit: Arduino Due [122] microcontroller	SVM	Accuracy: 99% (rotten/fresh)	Avocado fruits quality assessment
[123]	Sensor array: MQ-136, MQ-4, MQ-137, MQ-3, MQ-2, MQ-135, MQ-131, MQ-8, MQ-9; Signal processing unit: Raspberry Pi computer	PCA + KNN	Accuracy: 92% (spoiled/not spoiled)	Fruits (banana, pechay, carrot, grape) quality assessment
[124]	Sensor array: eight BME688 gas sensors [125]; Signal processing unit: Adafruit Huzzah32 (ESP32) development board [126]	Neural Networks [127]	Accuracy: 76% (spoiled/not spoiled)	Fruits and vegetables quality assessment
**Electronic Nose Systems for Spices’ Quality and Safety**				
[128,129]	Sensor array: TGS800, TGS813, TGS823, TGS2602, TGS2610, TGS2611, TGS2620, MQ-135; Signal processing unit: ADCs of a Programmable Interface Controller (PIC) microcontroller	Random Forest	Accuracy: 100% (nutmeg/ clove/cinnamon)	Identification of nutmeg, clove, and cinnamon
[130]	Sensor array: TGS2600, TGS2602, TGS2610, TGS813, TGS822, MQ-138, MQ-2 MQ-8; Signal proc. unit: AD7606 analog-to- digital data acquisition system [131] and S3C6410-based Linux platform [132]	PCA + SVM	Accuracy: 95%	Authenticity of star anise
**Electronic Nose Systems for Oils’ Quality and Safety**				
[133]	Sensor array: eight TGS and MQ sensors, and one temperature and relative humidity sensor; Signal proc. unit: RPi computer	Clustering technique [134]	Identification of three classes of palm oil: never heated/heated for 10 to 30 h/heated for 40 to 60 h	Palm oil quality assessment
[135]	Sensor array: MICS-6814 MOS sensor [136], MCP9700 temperature sensor [137]; Signal processing unit: ADS1015, PIC18F45K22, FT230XS USB/UART converter	ANNs	Accuracy: 99.49% (degummed/extraction/filtered/marketed)	Sunflower oil quality assessment
[138]	Sensor array: MQ-3, MQ-4, MQ-7, MQ-8, MQ-135, MQ-137, MQ-138, MG-811; Signal processing unit: N/A	ANNs (classification)	Accuracy: 86.5%	Extra-virgin olive oil quality assessment
		ANNs (regression)	Correlation coefficient: 0.93; Slope 0.90	
[139]	Sensor array: MQ-2, MQ-3, MQ-4, MQ-5, MQ-7, MQ-8, MQ-9, MQ-135; Sensor processing unit: Arduino Nano microcontroller	Discrete Fourier transform data analysis [140]	Accuracy: 91% (extra virgin/virgin); Accuracy: between 67% and 77% (extra virgin/virgin/blend /pomace/fresh air)	Olive oil quality assessment
[141]	Sensor array: MQ-3, TGS822, MQ-136, MQ-9, TGS813, MQ-135, TGS2602, TGS2620; Sensor processingunit: N/A	PCA	Total variance of the data for distilled water extracts from mint plants: 95%; Total variance of the data for mint essential oil: 89%	Mint essential oil and mint distilled water extracts quality assessment
		LDA	Accuracy for the classification of mint essential oil: 91.33%; Accuracy for mint distilled water extracts: 86.67%	
		ANNs	Accuracy for the classification of distilled water extracts 100%; Accuracy for the classification of mint essential oil 96.7%	
[142]	Sensor array: MQ-9, MQ-4, MQ-135, MQ-8, TGS2620, MQ-136, TGS813, TGS822, MQ-3; Signal proc. unit: N/A	PCA	Accuracy: 98%	Identification of essential oils from herbs and fruits
		LDA and Quadratic Discriminant Analysis	Accuracy: 100% (essential oil emissions from herbal leaves/fruits); Accuracy: 100% for Quadratic Discriminant Analysis and 98.9% for LDA (mango/lemon/orange /mint/tarragon/thyme)	
		SVM	Accuracy: 100% (essential oil emissions from herbal leaves/fruits); Accuracy: 98.9% (mango/lemon/orange /mint/tarragon/thyme)	
[143]	Sensor array: six different polymeric gas sensors (polymeric nanocom- posites of polyaniline with multiwalled carbon nanotubes and graphene oxide); Signal processing unit: N/A	PCA	Accuracy: 99.85%	Authenticity of clove oil
		Interactive Document Map multivariate projection techniques	Accuracy: 99.81%	
		LDA	Accuracy: 98.30%	
**Electronic Nose Systems for Coffee and Tea Quality and Safety**				
[144]	Sensor array: MQ-7, MQ-3, MQ-135, TGS2600, TGS2602, TGS2610, TGS2611, TGS2620, DHT22; Signal processing unit: N/A	Extreme Gradient Boosting [145]	Accuracy rates between 82% and 95% (sixteen classes of coffee)	Authenticity of coffee
		SVM	Accuracy rates between 81% and 95% (sixteen types of coffee)	
		CNN	Accuracy rates between 86% and 98% (sixteen types of coffee)	
		CNN + Long Short-Term Memory (LSTM) [146]	Accuracy rates between 83% and 98% (sixteen types of coffee)	
[147]	Sensor array: carbon nanotube-based multichannel with 64 interdigital electrodes; Sensor processing unit: N/A	LDA	Accuracy: 97.4% (three different coffee aromas)	Authenticity of coffee
[148]	Sensor array: four sensors (SnO2_bs1, ZH0504, SnO2 Au_bs2, SU0303) and two nanowire sensors (Sn-NW1, Sn-NW2); Sensor processing unit: N/A	PCA	N/A (four classes of roasted coffee beans)	Analysis of different methods of coffee roasting
[149]	Sensor array: TGS821, TGS2444, TGS823, TGS2600, TGS2602, TGS2610, TGS826, TGS2620; Signal processing unit: NI DAQ card, USB-6009	PCA	Accuracy: 95% (four acidity levels of coffee drinks)	Coffee drinks quality assessment
		Radial Basis Function Neural Network [150]	Accuracy: 94.75% (to predict the scores of acidity level)	
[151]	Sensor array: six sensing units (nanocomposites that stem from the combination of ZnO, In_2_O_3_, and ZnO/In_2_O_3_ nanoparticles with polypyrrole and poly(styrenesulfonate)); Signal processing unit: N/A	PCA + Euclidean distances by dendrograms	N/A (seventeen classes of coffee)	Authenticity of coffee
[152]	Sensor array: eight BME688 sensors; Signal processing unit: Adafruit Huzzah32 (ESP32) development board	Random Forest	MSE: 0.062	Authenticity of coffee
		Stochastic Gradient Descent [153]	Accuracy: 70.10% (two classes of coffee)	
		Adam Optimizer [154]	Accuracy: 67.70% (two classes of coffee)	
[155]	Sensor array: TGS832, TGS823, TGS2600, TGS2610, TGS2611; Signal processing unit: PCI6035E data acquisition card [156]	Bayesian classification [157]	Classification error in percentage 30.91% (four classes of tea)	Black tea quality assessment
**Electronic Nose Systems for Diary Quality and Safety**				
[158]	Sensor array: TGS2600, TGS822, TGS2611, TGS826, TGS2602, TGS832, TGS2620; Signal processing unit: Arduino microcontroller	PCA + LDA + SVM	Accuracy: 85%	Identification of milk source
		PCA + LDA + Logistic Regression [159]	Accuracy: 81.50%	
		PCA + LDA + Random Forest	Accuracy: 80.50%	
**Electronic Nose Systems for Alcoholic Beverage Quality and Safety**				
[160]	Sensor array: MQ-2, MQ-135, TGS825, WSP-2110, MP-503, TGS2602, WSP-1110, MQ-138, MQ-137, MQ-136; Sensor processing unit: N/A	Convolution Dot-Product Attention Mechanism [160], Residual network (ResNet50 mode) [161]	Accuracy: 98.47% (ten production origins of rice wines)	Identification of the origins of rice wines
[162]	Sensor array: TGS2600, TGS2602, TGS2603, TGS2610, TGS2611, TGS2620, TGS813, TGS822; Sensor processing unit: N/A	LDA + PCA + CNN-LSTM	Accuracy: 98% (whiskey/brandy /gin/vodka/tequila)	Identification of various types of spirit samples
[163]	Sensor array: TGS2600, TGS2603, TGS2610D, TGS2611C, TGS2620; Signal processing unit: N/A	Linear Discriminant	Accuracy: 69.23% (six brands of whiskey); Accuracy: 100% (whiskey regions of origin)	Authenticity of whiskey
		SVM	Accuracy: 82.05% (six brands of whiskey); Accuracy: 98.72% (whiskey regions of origin)	
		KNN	Accuracy: 61.54% (six brands of whiskey); Accuracy: 92.31% (whiskey regions of origin)	
		Bagged Tree [91]	Accuracy: 74.36% (six brands of whiskey); Accuracy: 94.87% (whiskey regions of origin)	
		Subspace Discriminant [164]	Accuracy: 70.51% (six brands of whiskey); Accuracy: 100% (whiskey regions of origin)	
[165]	Sensor array: TGS2600, TGS2602, TGS2603, TGS2610, TGS2611, TGS2620, TGS813, TGS822, DHT22; Signal processing unit: N/A;	CNN-LSTM	Accuracy: 93% (three whiskey types)	Authentication of whiskey
		CNN	Accuracy: 91% (three different types of whiskey)	
		LSTM	Accuracy: 91% (three different types of whiskey)	
		Recurrent Neural Networks [166]	Accuracy: 89% (three whiskey types)	
[167]	Sensor array: eight MOS sensors with two different types of copper oxide heterojunctions, ZnO–CuO and NiO–CuO; Signal processing unit: N/A	Hierarchical Clustering Analysis [168]	Euclidean distance: 0.5 (four samples of Chinese Jing Wine)	Identification of the same liquors manufactured in different years
[169]	Sensor array: TGS825, TGS821, TGS826, TGS822, TGS842, TGS813, TGS2610, TGS2201; Signal processing unit: N/A	PCA + Signal-to-Noise Ratio	First two principal components captured 92.47% of data variance (thirteen varieties of Chinese liquor)	Identification of several types of liquors
[170]	Sensor array: MQ-3, MQ-6, MQ-9, MQ-135, MQ-136, MQ-137, MQ-138, MQ-139, SHT15 [171]; Signal processing unit: NI DAQ card, USB-6009	PCA + Multi-Layer Perceptron (MLP) [172]	Accuracy: 100% (three distinct local Thai spirits)	Identification of local Thai craft spirits
		PCA + k-Means	Accuracy: 72.23% (three distinct local Thai spirits)	
[173]	Sensor array: MQ-3, MQ-4, MQ-7, MQ-8, MQ-135, MQ-136, MQ-137, MQ-138, MG811 [174], AM2320 [175]; Signal processing unit: microcontroller with an onboard ADC	ANNs	Correlation coefficient: 0.97 (to predict seventeen volatile aromatic compounds)	Beer quality assessment

**Table 6:** Summary of gas sensors used in e-nose systems and their key features (response time—the time a sensor takes to reach a certain percentage of its final output signal after exposure to a target gas; resume time—the time the sensor takes to return to its baseline signal after the removal of the target gas).

#	Sensor	Target Gas	Detection Range [ppm]	Response and Resume Time [s]	Heater Consumption [mW]	Preheat Time [min/h/day]
1	BME688	IAQ, bVOC, eCO_2_ bVOC: (5 ppm Ethane, 10 ppm Isoprene/2-methyl-1,3 Butadiene, 10 ppm Ethanol, 50 ppm Acetone, 15 ppm Carbon monoxide)	0–500 (IAQ), bVOC, CO_2_ P: 300–100 hPa, H: 0–100% T: −40–85 °C	1/3/300	0.16–21.6	30 min
2	MQ-2	Flammable gas, smoke	300–10,000 ppm (Flammable gas)	60	950	48 h
3	MQ-3	Alcohol, Benzine	0.05–10 mg/L Alcohol	-	750	24 h
4	MQ-4	Methane	300–10,000 ppm (CH_4_)	60	950	48 h
5	MQ-5	Liquefied petroleum gas, Methane	300–10,000 ppm (CH_4_, C_3_H_8_)	60	950	48 h
6	MQ-6	Liquefied petroleum gas	300–10,000 ppm (Propane)	60	950	48 h
7	MQ-7	Carbon monoxide	20–2000 ppm (CO)	60	350	48 h
8	MQ-8	Hydrogen gas	100–1000 ppm (H_2_ gas)	60	950	48 h
9	MQ-9	Carbon monoxide and Combustible gas (Methane and Liquefied petroleum gas)	10–1000 ppm (CO) 100–10,000 ppm (Combustible gas)	60	350	48 h
10	MQ-131	Ozone	10–1000 ppm (O_3_)	110	950	48 h
11	MQ-135	Ammonia gas, Sulfide, Benzene series steam	10–1000 ppm (Ammonia gas, Toluene, Hydrogen, smoke)	60	950	48 h
12	MQ-136	Hydrogen sulfide gas	1–200 ppm (H_2_S gas)	60	950	48 h
13	MQ-137	Ammonia gas	5–500 ppm (NH_3_ gas)	60	900	48 h
14	MQ-138	Toluene, acetone, alcohol, hydrogen	5–500 ppm	60	900	48 h
15	MQ-139	Freon	10–1000 ppm	180–300	900	48 h
16	ZE03-NH_3_	CO, O_2_, NH_3_, H_2_S, NO_2_, O_3_, SO_2_, CL_2_, HF	0–1000 ppm (CO), 0–25% vol (O_2_), 0–100 ppm (NH_3_), 0–100 ppm (H_2_S), 0–20 ppm (NO_2_), 0–10 ppm (HF), 0–20 ppm (SO_2_), 0–10 ppm (CL_2_), 0–20 ppm (O_3_)	15–150	20	-
17	ZE03-C_2_H_4_	CO, O_2_, NH_3_, H_2_S, NO_2_, O_3_, SO_2_, CL_2_, HF, H_2_, PH_3_, HCL, C_2_H_4_	-	15–150	20	-
18	MICS-6814	Carbon monoxide, Nitrogen dioxide, Ethanol, Hydrogen, Ammonia, Methane, Propane, Iso-butane	1–1000 ppm (CO), 0.05–10 ppm (NO_2_), 10–500 ppm (C_2_H_5_OH), 1–1000 ppm (H_2_), 1–500 ppm (NH_3_), CH_4_ > 1000 ppm, C_3_H_8_ > 1000 ppm, C_4_H_1_0 > 1000 ppm	-	43–76	-
19	TGS2201	Diesel exhaust, Gasoline exhaust	0.1–10 ppm (NO, NO_2_) 10–1000 ppm (CO, H_2_, HC)	-	505	7 d
20	TGS2444	Ammonia gas, Hydrogen sulfide gas, Ethanol	10–300 ppm of NH_3_, 10–100 ppm of H_2_S, 300–1000 ppm of Ethanol	60–180	56	48 h
21	TGS2600	Hydrogen, Ethanol	1–30 ppm of H_2_	-	210	7 d
22	TGS2602	VOCs, Ammonia, Hydrogen sulfide gas	1–30 ppm of EtOH	-	280	7 d
23	TGS2603	Trimethylamine, Methyl mercaptan	1–30 ppm of EtOH	-	240	96 h
24	TGS2610	Butane, Liquefied petroleum gas	1–25 % LEL	-	280	7 d
25	TGS2611	Methane, Natural gas	500–10,000 ppm	-	280	7 d
26	TGS2612	Methane, Propane, Iso-butane	1–25 % LEL of each gas	-	280	7 d
27	TGS2620	Alcohol, Organic solvent vapors	50–5000 ppm EtOH	-	210	7 d
28	TGS800	General air contaminants	1–30 ppm	-	660	-
29	TGS813	Combustible gases	500–10,000 ppm of Methane	-	835	-
30	TGS821	Hydrogen	30–1000 ppm of H_2_	-	660	-
31	TGS822/823	Alcohol, Organic solvents	50–5000 ppm of Ethanol	-	660	-
32	TGS825	Hydrogen sulfide gas	5–100 ppm of (H_2_)	-	660	-
33	TGS826	Ammonia gas	30–300 ppm of NH_3_	-	835	-
34	TGS832	R-134a	100–3000 ppm of R-134a	-	835	-
35	TGS842	Methane natural gas	500–10,000 ppm of CH_4_	-	835	-
36	TGS880	Fumes from food (alcohol, odor)	10–1000 ppm (Air and Ethanol)	-	835	-
37	WSP1110 Obsolete	NO_2_ sensor	0.1–10 ppm NO_2_	-	-	-
38	WSP2110	Toluene, Methanal, Benzene, Alcohol, Acetone	1–50 ppm NO_2_	70	300	120 h
39	MP503	Alcohol, Smoke, Iso-butane, Methanal	10–1000 ppm (Alcohol)	60	300	48 h
40	MG811	Carbon dioxide	350–10,000 ppm (CO_2_)	20	1200	-

Note: No documentation was found for the MQ-1, HGS1000, HGS1001, HGS1002, and HGS1007 sensors; therefore, they are not included in this table.

**Table 7:** Summary of commercial electronic nose systems for food quality and safety.

Commercial E-Nose	Paper	Data Analysis Techniques	Evaluation and Performance Metrics	Application Area
PEN3: A sensor array of ten different metal oxides single thick film sensors [177]	[183]	Recurrent Criss-Cross Attention Network [184]	Accuracy: 98%	Peanuts quality assessment
	[185]	Statistical analysis on data collected by PEN3 (weight loss measurements and firmness analysis also performed)	Prove that ilmenite-grafted graphene oxide coating reduces postharvest losses	Postharvest preservation of fruits (bananas)
	[186]	PCA (e-nose and Headspace-Gas Chromatography-Ion Mobility Spectrometry)	Accuracy: 100% (genuine/fake)	Amomi fructus authenticity
		Partial Least Squares-Discriminant Analysis (PLS-DA) [187] (e-nose and Headspace-Gas Chromatography-Ion Mobility Spectrometry)	Accuracy: 97.96% (origin identification)	Amomi fructus origin identification
	[188]	PCA + ANNs	Accuracy: 99%	Milk safety assessment
	[189]	Solid-Phase Microextraction [190] coupled with GC–MS and e-nose analysis	Not discussed	Development of structured lipids with enhanced flavor profiles for dairy products and functional food
	[191]	Dung Beetle Optimizer algorithm [192] combined with 10 different machine learning methods	Coefficient of determination > 0.895	Prediction of the electronic sensory characteristics of fermented milk
	[193]	Proposed data augmentation model (e-nose + e-tongue) + CNN	Accuracy: 95.34% (four types of mixed solution); Accuracy: 97.78% (five brands of beer); Accuracy: 97.37% (five kinds of apple)	Quality of different food
Fox 3000: Two sensor chambers equipped with twelve MOS sensors [178]	[178]	Random Forest	Accuracy: 95.30%	Mandarin orange quality assessment
Fox 4000: An injection system, sensor chambers with eighteen MOS sensors, a mass flow controller, and a micro-controller acquisition board [179]	[194]	PCA	Discrimination index: 93 (seven batches of hydrolysate)	Quality of baked goods (effects of enzymatic hydrolysis on soy protein concentrate)
	[195]	PCA	PCA1: 94.54%, PCA2: 3.38% of the total variance (untreated sample/pasteurized sample/treated sample/sterilized sample in 0, 30 and 60 days of storage)	Shelf life of chicken products quality assessment
	[196]	PCA	Discrimination index: 90 (eight types of plum jam samples)	Evaluation of the characteristics of sugar-free plum jams
	[197]	PCA + CA + Partial Least Squares regression [198] (GC–MS and e-nose data)	Correlation coefficients > 0.98 (for 14 characteristic aroma-active compounds)	Mitten crab quality assessment
FOODsniffer [180]	[199]	E-nose data analysis compared with microbiological and GC–MS analyses	FOODsniffer can anticipate the unacceptability conditions of salmon (at 22 °C, 10% of samples are `Not satisfactory’ when FOODSniffer is `Green’)	Salmon fillet and burger quality and safety assessment
	[200]	E-nose data analysis (PCA) compared with physicochemical measurements of meat quality	PC1–71.13%, PC2: 83.70% of total variance	Meat quality assessment
NeOse Pro: A gold-layer-based optoelectronic sensor array featuring sixty-three non-specific peptides [181]	[201]	PCA + Gas Chromatography with Ion Mobility Spectrometry (GC–IMS)	Completely separate one sample	Plant-based beverage quality assessment
		PCA + e-nose	Completely separate seven samples	
		LDA + GC–IMS	Accuracy between 15.4% and 100%	
		LDA + e-nose	Accuracy between 96.2% and 100%	
Cyranose^@^ 320: An array of thirty-two nanocomposite sensors [182]	[202]	Proposed e-nose pattern recognition algorithm	Accuracy: 80% at room temperature	Identification of *Terfezia arenaria* truffle

**Table 8:** Summary of electronic noses developed by academic research groups for food quality and safety.

E-Nose	Paper	Data Analysis Techniques	Evaluation and Performance Metrics	Application Area
LibraNose: An array of eight QCM non-selective sensors coated with different polypyrrole derivatives—University of Rome Tor Vergata, Italy [203]	[209]	E-nose data analysis (PCA + Random Forest regression)	Accuracy: 92.8% (for predictions of *B. thermosphacta*)	Meat quality assessment
		High Performance Liquid Chromatography + Random Forest regression	Accuracy: 100% (for predictions of *Lactobacilli*)	
		GC–MS + Random Forest regression	Accuracy: 93.9% (for predictions of *Enterobacteriaceae*)	
		GC–MS + kNN-R	Accuracy: 96.0% (for predictions of *Pseudomonads*)	
	[210]	PCA + Proposed data model based on Adaptive Fuzzy Logic System	Accuracy: 94.28% (fresh/semi-fresh/spoiled)	Monitoring of meat spoiling during storage
	[211]	PCA + Proposed Multi-Input Multi-Output Clustering-based Fuzzy Wavelet Neural Network model	Accuracy: 95.71% (fresh/semi-fresh/spoiled); RMSE: 0.2969 to predict the microbial load on meat surface	Meat quality assessment
	[212]	Proposed model based on ensemble-based (Bagging and Boosting) SVM	Accuracy: 84.1% (fresh/semi-fresh/spoiled)	Meat quality assessment
		Proposed model based on ensemble-based (Bagging and Boosting) SVM-regression	Accuracy: 85% to predict bacterial species counts	
E-nose with an array of twelve QCM sensors—University of Rome Tor Vergata, Italy [204]	[204]	PLS-DA	94% of the original data’s variation can be represented in a reduced-dimensional space; Accuracy: 100% (five different classes of sparkling wines)	Identification of rosé sparkling wines
	[213]	PLS-DA	85% of the original data’s variation can be represented in a reduced-dimensional space; Accuracy between 60% and 100% for the first stages of *Botrytis cinerea* infection (1, 2, 3 days)	Identification of noble rot (a fungus also known as *Botrytis cinerea*) in postharvest wine grapes
E-nose with an array of eight QCM sensors—University of Rome Tor Vergata, Italy [205]	[205]	PCA + LDA	Accuracy: 71.4% (*Aspergillus niger*/ *Aspergillus fumigatus*/ *Aspergillus flavus*)	Identification of *Aspergillus* Species
E-nose with four gas sensors (BME680 [214], SGP30 [215], CCS811 [216], iAQ-Core [217]) —Industrial Engineering School of the University of Extremadura, Spain [206]	[206]	E-nose data analysis (PCA + PLS-DA) compared with GC–MS	PC1–83.5%, PC2–12.3% of the total variance; Accuracy: 100% (six classes of roasted coffee beans exposed to different heat treatment conditions)	Roasted coffee quality assessment
	[218]	PCA + PLS-DA	Whole roasted almonds–R^2^: 0.89 for acrylamide and furfural, R^2^: 0.83 for hydroxymethylfurfural Ground roasted almonds–R^2^: 0.99 for acrylamide, R^2^: 0.98 for hydroxymethylfurfural, R^2^: 0.88 for furfural	Prediction of contaminants in roasted almonds
Agrinose: An array of eight MOS sensors (AS-MLV-P2 [219], TGS2602, TGS2600, TGS2603, TGS2610, TGS2611, TGS8100 [220], TGS2620) —Institute of Agrophysics IA PAS, Poland [207]	[221]	Proposed model based on a three-parameter method based on the impregnation time, cleaning time, and maximum response of chemically sensing sensors + PCA compared with GC–MS	PC1 + PC2 describe 72.64% of the total variance and enable clear separation of different sample classes	Assessment of the suitability of bread for consumption after storage
	[222]	Proposed model based on a three-parameter method based on the impregnation time, cleaning time, and maximum response of chemically sensing sensors + PCA compared with Fourier Transform Infrared Spectroscopy [223] and GC–MS	PC1 + PC2 describe 79.25% of the total variance and enable clear separation of different sample classes	Identification of rapeseed spoilage
E-nose with nine MOS sensors (MQ-2, MQ-3, MQ-4, MQ-5, MQ-6, MQ-7, MQ-8, MQ-9, MQ-135)—Department of Biosystems Engineering, Bu-Ali Sina University, Iran [208]	[224]	PCA	Included 55%, 75%, 47%, and 53% of data for unprocessed whole/dried slices/powder/tablet	Garlic quality assessment
		LDA	Accuracy: 90%, 93.33%, 88.89%, 60% (unprocessed whole/dried slices/powder/tablet)	
		SVM	Accuracy: 72.22%, 80.00%, 75.55%, 40% (unprocessed whole/dried slices/powder/tablet)	
		Backpropagation Neural Network [225]	Accuracy: 100%, 97.80%, 92.2%, 77.78% (unprocessed whole/dried slices/powder/tablet)	

**Table 9:** Summary of gas sensors used in e-nose systems and their key features.

#	Sensor	Target Gas	Detection Range [ppm]	Response and Resume Time [s]	Heater Consumption [mW]	Preheat Time [min/h/Day]
1	BME688	IAQ, bVOC, eCO_2_ bVOC: (5 ppm Ethane, 10 ppm Isoprene/2-methyl-1,3 Butadiene, 10 ppm Ethanol, 50 ppm Acetone, 15 ppm Carbon monoxide)	0–500 (IAQ), bVOC, CO_2_ P: 300–1100 hPa, H: 0–100% T: −40–85°C	1/3/300	0.16–21.6	30 min
2	SGP30 End of life	VOC, eCO_2_, Ethanol, Hydrogen sulfide gas	0–60,000 ppb (VOC), 400–60,000 ppm (eCO_2_) 0– 1000 ppm (Ethanol, H_2_S)	1	86.4	24 h
3	CCS811	TVOC, eCO_2_	0–1187 ppb (TVOC), 400–8192 ppm (eCO_2_)	0.25/1/10/60	1.2–46	48 h
4	iAQ-Core Obsolete	eCO_2_, TVOC	450–2000 ppm (eCO_2_), 125–600 ppb (TVOC )	1/11	9–69	-
5	AS-MLV-P2 Obsolete	CO, Butane, Methane, Ethanol, Hydrogen	30–500 ppm (CO), 15–150 ppm (Butane), 250–4500 ppm (CH_4_), 10–200 ppm (Ethanol), 25–500 ppm (H)	1/10	50	5 d
6	TGS8100	Methane, Iso-butane, CO, Hydrogen, Ethanol	1–100 ppm, 1–30 ppm (H_2_)	-	15	>1 h

**Table 10:** Summary of new gas sensors for the food industry.

Reference and Sensor Type/Technology	Target Compounds/ Application	Data Analysis Techniques	Key Features/Results
[226]: Silicon NanoWires + multi-walled carbon nanotube	Essential oils, alcoholic beverages, general food	PCA	Fast response time (20–30 s), high selectivity, dual surface, and chemical modification
[227]: Graphene junctions	Aflatoxin B1	N/A	1.2 V bias yields >3 µA current change; suitable for rapid e-nose integration
[228]: Plasmonic arrays + chemometrics + machine learning	Multiple VOCs in food	PCA + LDA	Uses Surface-Enhanced Raman Spectroscopy, mimics animal olfaction; machine learning enables multi-analyte detection
[229]: Memristor-based in-memory computing + MOS sensor array	Various gases (15 sensors)	CNN	94% classification accuracy; 20.2 mW power; fast response time (<0.4 ms inference time); compact processing scheme
[230]: Graphene + Metal Phthalocyanines	Ammonia gas, interfering gases	PCA	5-sensor array (Co-Pc, Ni-Pc, Zn-Pc, Fe-Pc, pristine); promising for food quality monitoring
[231]: Film Bulk Acoustic Resonator sensors	General gases; example: banana freshness	Real-time signal processing and pre-processing + Discriminative analysis	Miniaturized portable e-nose; 6–8× more sensitive than polymer-coated Film Bulk Acoustic Resonator; drift-compensated
[232]: Colorimetric Fe(II) complex	Ammonia gas	PCA + Hierarchical cluster analysis, SVM	Detects 105 ppb at room temp; reusable; no external energy needed
[233]: CNT + olfactory receptor (ODR-10)	Diacetyl in alcoholic beverages	Sensitivity and selectivity analysis	Detection limit of 10 fM; better than fluorescence assays and GC–IMS in classification

**Table 11:** Summary of datasets used in e-nose research for food quality and safety.

Dataset	Description	Paper	Data Analysis Techniques	Evaluation and Performance Metrics
[234,235]	2220 sensor signal responses collected from twelve cuts of beef samples in four different degrees of freshness using eleven gas sensors	[236]	Proposed model based on 1D-CNN	Accuracy: 97.22% (excellent/good/ acceptable/spoiled)
		[237]	ANNs	Accuracy: 99.9% (excellent/good/ acceptable/spoiled)
			Linear Regression	Accuracy: 98.9% (excellent/good/ acceptable/spoiled))
			KNN	Accuracy: 98.8% (excellent/good/ acceptable/spoiled)
		[238]	Proposed MLP model on Field Programmable Gate Array	Accuracy: 92.72% (excellent/good/ acceptable/spoiled)
		[239]	Proposed approach based on Single Plurality Voting System model + Decision Tree	Accuracy: 91.13% (excellent/good/ acceptable/spoiled)
			Proposed approach based on Single Plurality Voting System model + KNN	Accuracy: 88.69% (excellent/good/ acceptable/spoiled)
			Proposed approach based on Single Plurality Voting System model + LDA	Accuracy: 80.73% (excellent/good/ acceptable/spoiled)
[240]	420 samples for seven different mixtures of beef and pork collected from eight gas sensors	[241]	Proposed model based on a conventional Deep Extreme Learning Machine with an autoencoder for feature learning	Accuracy: 99.85% (seven combination mixtures of meat)
			Proposed model based on SVM with a Radial Basis Function kernel	Accuracy: 93.48% (seven combination mixtures of meat)
			Proposed model based on a conventional Deep Extreme Learning Machine with PCA for feature learning	Accuracy: 99.97% (seven combination mixtures of meat)
			Proposed model based on PCA + SVM with a Radial Basis Function kernel	Accuracy: 96.88% (seven combination mixtures of meat)
[242,243]	Time series data for 235 wine samples collected from six gas sensors	[244]	Proposed model based on CNN	Accuracy: 99.2% (low quality/average quality/high quality)
[245]	48,846 rows for rice quality acquired from nine gas sensors and four other sensors for related data	[245]	Proposed model based on KNN	R^2^: 0.7217; RMSE: 3.8043
		[246]	Gradient Tree Boosting	Accuracy: 96% (expired/non-expired)
		[247]	Complement Naïve Bayes classifier	Accuracy: 98% (expired/non-expired)
			Multinomial Naïve Bayes classifier	Accuracy: 97% (expired/non-expired)
			Gaussian Naïve Bayes classifier	Accuracy: 82% (expired/non-expired)
			Bernoulli Naïve Bayes classifier	Accuracy: 52% (expired/non-expired)
		[248]	MLP	Accuracy: 99.84% (expired/non-expired)

**Table 12:** Summary of gaps and corresponding solutions in this work.

Identified Gap	Recommended Solution in This Review
Low real-world deployment despite high lab accuracy	Provide case studies and benchmarking tables to bridge lab-to-field gaps
Sensor selectivity and sensitivity challenges	Develop gas-sensitive materials with enhanced selectivity and sensitivity for food VOCs, or design bio-inspired or biomimetic sensors that mimic natural senses to improve detection accuracy in food analysis
Sensor response time challenges	Design gas sensors with fast response times
Sensor drift and calibration challenges	Introduce adaptive/recalibrating machine learning models and emphasize real-time feedback control
Black-box nature of machine learning models used in classification	Recommend interpretable machine learning models and alignment with food safety regulations (i.e., Codex/ISO)
Lack of efficient multi-sensor (e-nose, e-tongue, e-eye) data fusion algorithms results in incomplete food profiling	Develop advanced data fusion frameworks using machine learning, hybrid fusion techniques, and synchronized pre-processing
Miniaturization and portability challenges	Recommend integration of micro-electro-mechanical systems/ nano-electro-mechanical systems technologies for compact, low-power devices
Lack of standardization in methodology and validation	Recommend universal protocols for data collection, validation, and sensor benchmarking
Lack support in user-friendly visualization of the odor classification and identification results	Introduce real-time data analysis platforms for on-site decision making

## Abbreviations

The following abbreviations are used in this manuscript:

ADC	analog-to-digital converters
ANNs	artificial neural networks
CNN	Convolutional Neural Network
CNT	carbon nanotube
CP	conducting polymer
GC–IMS	Gas Chromatography with Ion Mobility Spectrometry
GC–MS	Gas Chromatography–Mass Spectrometry
KNNs	k-nearest neighbors
LDA	linear discriminant analysis
LSTM	Long Short-Term Memory
MLP	Multilayer Perceptron
MOS	metal oxide semiconductor
MSE	Mean Squared Error (MSE)
PCA	principal component analysis
PIC	Programmable Interface Controller
PLS-DA	Partial Least Squares-Discriminant Analysis
PNN	Probabilistic Neural Network
PPM	Parts per Million
QCM	quartz crystal microbalance
R^2^	R-squared
RMSE	Root Mean Square Error
SAW	surface acoustic wave
SVMs	support vector machines
SVR	Support Vector Machine Regression Technique
VOCs	volatile organic compounds
WoS	Web of Science
